# Molecular Characterization of Advanced Colorectal Cancer Using Serum Proteomics and Metabolomics

**DOI:** 10.3389/fmolb.2021.687229

**Published:** 2021-07-27

**Authors:** Jun Rao, Xianghui Wan, Fangfang Tou, Qinsi He, Aihua Xiong, Xinyi Chen, Wenhao Cui, Zhi Zheng

**Affiliations:** ^1^ Jiangxi Cancer Hospital, Jiangxi Cancer Hospital of Nanchang University, Nanchang, China; ^2^ Department of Hematology and Oncology, Beijing University of Chinese Medicine, Beijing, China; ^3^ Department of Pharmacology, Kyoto Prefectural University of Medicine, Kyoto, Japan

**Keywords:** colorectal cancer, DIA-MS, UPLC/Q-TOF-MS/MS, correlation analysis, biomarker

## Abstract

Colorectal cancer (CRC) is a growing public health concern due to its high mortality rate. Currently, there is a lack of valid diagnostic biomarkers and few therapeutic strategies are available for CRC treatment, especially for advanced CRC whose underlying pathogenic mechanisms remain poorly understood. In the present study, we investigated the serum samples from 20 patients with stage III or IV advanced CRC using data-independent acquisition (DIA)-based proteomics and ultra-performance liquid chromatography coupled to time-of-flight tandem mass spectrometry (UPLC-TOF-MS/MS) metabolomics techniques. Overall, 551 proteins and 719 metabolites were identified. Hierarchical clustering analysis revealed that the serum proteomes of advanced CRC are more diversified than the metabolomes. Ten biochemical pathways associated with cancer cell metabolism were enriched in the detected proteins and metabolites, including glycolysis/gluconeogenesis, biosynthesis of amino acids, glutathione metabolism, and arachidonic acid metabolism, etc. A protein-protein interaction network in advanced CRC serum was constructed with 80 proteins and 21 related metabolites. Correlation analysis revealed conserved roles of lipids and lipid-like molecules in a regulatory network of advanced CRC. Three metabolites (hydroquinone, leucenol and sphingomyelin) and two proteins (coagulation factor XIII A chain and plasma kallikrein) were selected to be potential biomarkers for advanced CRC, which are positively and significantly correlated with CEA and/or CA 19–9. Altogether, the results expanded our understanding of the physiopathology of advanced CRC and discovered novel potential biomarkers for further validation and application to improve the diagnosis and monitoring of advanced CRC.

## Introduction

Colorectal cancer (CRC) is the third most common malignancy and the second leading cause of cancer-related death worldwide (Bray et al., 2018; Cantor et al., 2020). It is estimated that by the year of 2030, CRC may account for one in every ten cancer cases and deaths, and increase the global health burden by 60% (Bray et al., 2018). The high mortality rate of CRC is mainly due to its late diagnosis when CRC is already in advanced stages and metastasis has already occurred. Only 9% of CRC patients are practically diagnosed at stage I, and most (91%) are diagnosed at stage II, III or IV (Hammond et al., 2016). Well documented risk factors of CRC include cigarette smoking, physical inactivity, obesity, and high consumption of alcohol or red meat (Hissong and Pittman, 2020). Family history and certain medical conditions including inflammatory bowel disease are also associated with CRC (Xue et al., 2017).

The pathogenic mechanisms of CRC are complex and heterogeneous, and molecular changes in the tumor determine both the histologic type of premalignant lesion and the time to malignant transformation (Hissong and Pittman, 2020). Secondary to inactivation of the adenomatous polyposis coli (APC) tumor suppressor gene, chromosomal instability is a commonly characterized molecular event in CRC, which subsequently results in hyper-activation of the WNT signaling pathway (Fearon and Vogelstein, 1990). Another molecular event, the microsatellite instability, occurs in 15% of CRC (Hissong and Pittman, 2020). CRC also involves abnormalities in MLH-1, PMS-2, MSH-2, MSH-6, or POL-E gene which are all necessary for repairing DNA mismatches. In the past few years, genomic and transcriptomic landscapes of CRC have been investigated and many genomic alterations and extensive molecular heterogeneity of the disease have been identified (Cancer Genome Atlas Netwo, 2012; Vasaikar et al., 2019). For example, a genome-scale analysis of 276 CRC patients was conducted to characterize somatic alterations in CRC (Vasaikar et al., 2019). The results showed that 24 genes including APC, TP53, ARID1A, and SOX9 are significantly mutated, suggesting a number of new potential therapeutic strategies to CRC.

Recent advances in proteomics and metabolomics techniques have extended our understanding of pathways that control cell proliferation, differentiation, and death (Chen et al., 2019). Identification of changed proteins or metabolites in the development of CRC is important to the discovery of new potential biomarkers for early diagnosis (Ritchie et al., 2010). Identified proteins, metabolites and their corresponding pathways are attractive therapeutic targets for cancer treatment. Proteomics is a high-throughput large-scale approach that allows for simultaneous detection of thousands of proteins in many sample types such as cell, tissue, or body fluids. In 2016, Ward et al. employed surface-enhanced laser desorbtion/ionisation technique to characterize the serum proteomes of 62 CRC patients and 31 healthy individuals, and the study identified complement C3a des-arg, α1-antitrypsin and transferrin to have diagnostic potentials (Ward et al., 2006). Vasaikar et al. conducted a proteogenomic study on prospectively collected CRC tumor tissues and adjacent normal tissues (Vasaikar et al., 2019). An association between increased glycolysis in microsatellite instability-high (MSI-H) tumors and decreased CD8 T cell infiltration was identified, suggesting the glycolysis pathway could be a potential target to reverse the resistance of MSI-H tumors to immune check-point blockade treatment. Similar to proteomics, metabolomics is a large-scale high-throughput omics technology that enables comprehensive and semi-quantitative detection of a large number of metabolites in biological samples. Metabolomic studies in various cancers such as CRC, gastric cancer, liver cancer, and pancreatic cancer have demonstrated its great potentials in improving tumor diagnosis and therapy (Zheng et al., 2017; Fan et al., 2018; Zheng et al., 2018). For instance, Kim et al. performed urine-NMR metabolomics study on 92 patients with colorectal neoplasia and 156 healthy individuals to screen for advanced adenoma and stage 0 CRC (Kim et al., 2019), and the receiver operating characteristics curve analysis results revealed that 3-aminoisobutyrate, taurine, and alanine were good indicators of CRC.

For general CRC treatment, different strategies including surgery, radiation therapy, chemotherapy, targeted drug therapy, and immunotherapy have been adopted (Khiavi et al., 2019). For advanced-stage CRC, chemotherapy is commonly recommended, and targeted therapies including anti-epidermal growth factor receptor (anti-EGFR) agents are frequently used in combination with chemotherapy (Rawla et al., 2019; Wang et al., 2019). The treatment effects of invasive CRC will depend on tumor location, stage, and underlying molecular changes including genetic and metabolic alterations. Comprehensive molecular characterization studies of advanced CRC, particularly combined proteomic and metabolomic study, have been rare. In the current study, we conducted nontargeted DIA-MS proteomics and UPLC/Q-TOF-MS/MS metabolomics analyses on 20 serum samples from advanced CRC patients. The aim is to identify key regulatory elements (proteins and/or metabolites) and pathways in advanced CRC, which may serve to be potential biomarkers for early diagnosis and novel therapeutic targets of advanced CRC.

## Materials and Methods

### Study Participants

The study was approved by the Ethics Committee of Jiangxi Cancer Hospital and performed in accordance with the Declaration of Helsinki. Written informed consents were obtained from all participants. In total, 20 patients (N1—N20) diagnosed with advanced CRC at stage III or IV were recruited. The average age of the 20 patients was 51, ranging from 29 to 76. None of the participants had been diagnosed with other major chronicle diseases or cancers, and none had received any drug treatment in the previous 3 months before sampling. The demographic and clinical characteristics of the 20 patients were listed in Table 1.

**TABLE 1:** Characteristics of 20 CRC patients.

Characteristics	CRC patients
No. of subjects	20
Race	Han
Age (median)	51 years
Gender (%men)	60% (12/20)
UICC Stage	
III	15% (3/20)
IV	85% (17/20)
CA19-9 (ng/ml)	
Average	2000.60
≤37	50% (10/20)
>37	50% (10/20)
CEA (ng/ml)	
Average	503.41
≤5	55% (11/20)
>5	45% (9/20)

### Proteomic Analysis

The proteomic analysis of the serum samples was conducted using the combination of DIA and a data dependent acquisition (DDA)-based ion library as previously reported (Chen et al., 2019). Each sample of 2 μL serum was first diluted with a lysis buffer containing 100 mM Tris-HCL (pH 8.5, Sigma, MO, United States), 8 M Urea (Sigma, MO, United States), 1 mM EDTA, and 1 mM PMSF, and then centrifuged at 15000 *g* for 15 min at 4°C. The extracted proteins in the supernatant was quantified using a BCA protein assay kit (Bi Yuntian, Shanghai, China), digested in trypsin (Promega, Madison, WI) after reduction and alkylation using the FASP (filter aided sample preparation) method (Wisniewski et al., 2009). The concentration of digested peptides was determined by measuring the absorbance at 280 nm using a NanoDrop 2000 instrument (ThermoFisher Scientific, United States). Each 3 μg of trypsin-digested peptides was mixed with iRT peptides (Biognosys, Schlieren, Switzerland) and analyzed in the DDA mode on an Orbitrap Fusion Lumos mass spectrometer (ThermoFisher Scientific, United States) equipped with an EASY-nLC 1000 system (ThermoFisher Scientific, United States) (Chen et al., 2019). The peptides were separated on a 150 μm I.D. × 15 cm C18 trap column (C18, 1.9  μm, 120 Å, Dr Maisch GmbH) with a mobile solution flow rate of 600 nL/min. The gradient elution program was as the following: 7–20% solvent B for 80 min, 20–32% solvent B for 25 min, 32–90% solvent B for 13 min. Data was acquired with full scans (m/z 350–1500) at a mass resolution of 60,000 at m/z 200. The top 20 precursor ions were selected for fragmentation in the HCD (high energy collision dissociation) cell at normalized collision energy of 32%, and fragment ions were scanned at a resolution of 30,000 at m/z 200. The automatic gain control (AGC) was set to 4e5 for full MS with maximum ion injection time of 50 ms, and 5e4 for MS/MS with maximum ion injection time of 54 ms. The dynamic exclusion was 30 s.

The DIA analysis was performed the same as for DDA. The full scan in DIA analysis was at a resolution of 60,000 over m/z 350—1500, DIA scan resolution was 30,000, collision energy was 32%, AGC target was 5e^5^, and maximum injection time was 74 ms. There were 45 variable DIA windows set from 350 to 1500 m/z. Protein identification and quantification were performed using Spectronaut pulsar X 12.0 (Biognosys) with default setting. For protein identification, DDA raw files were searched against the human Uniprot fasta database, and three to six fragments with the highest quality were selected for each peptide to generate a spectral library. Peptide FDR (false discovery rate), PSM FDR, and protein FDR were all set to 1%. The iRT Calibration R square was set to 0.8. For protein quantification using the DIA data, RT regression type was set as Local (Non- Linear) Regression. All results were filtered by a Q-value cutoff of 0.01 (corresponds to a FDR of 1%). *p*-value estimator was performed by Kermel Density Estimator. Area was used for protein quantification. Every peptide was validated with at least three fragment-ions.

### Metabolomics Analysis

Metabolites in the serum samples were extracted with 120 μL of 50% methanol buffer (Chen et al., 2019). For global metabolomics analysis, an ultra-performance liquid chromatography (UPLC) system (SCIEX, Cheshire, United Kingdom) coupled to a high-resolution tandem mass spectrometer (Triple TOF 5600 plus; SCIEX) were used. An ACQUITY UPLC T3 column (100 mm × 2.1 mm, 1.8 µm, Waters, United Kingdom) was employed for reversed phase separation. The two mobile phase solutions were solvent A (water, 0.1% formic acid) and solvent B (Acetonitrile, 0.1% formic acid), the mobile phase solution flow rate was 0.4 ml/min. The gradient elution program was as the following: 0–0.5 min, 5% B; 0.5–7 min, 5–100% B; 7–8 min, 100% B; 8–8.1 min, 100–5% B; 8.1–10 min, 5% B. The injection volume for each sample was 4 µL. The Q-TOF was performed in both positive and negative ion modes (Chen et al., 2019). The ionspray voltage floating in positive and negative ion mode were set at 5000 and −4500 V, respectively. The XCMS software was used for MS data pretreatments including peak picking, peak grouping, retention time correction, second peak grouping, and annotation of isotopes and adducts. Online databases including Human Metabolome Database (HMDB) and Kyoto Encyclopedia of Genes and Genomes (KEGG) were employed for metabolite annotations. An in-house fragment spectrum library of metabolites was used for compound identification by MS^2^ matching. Five pooled quality control (QC) samples were prepared by combining 10 μL of each extraction and injected with the true samples for quality control purpose.

### Data Analysis

All of the raw mass spectrometry data have been deposited to the ProteomeXchange Consortium (http://proteomecentral.proteomexchange.org) *via* the iProX partner repository and the dataset identifier is PXD025041. Both the proteomic and metabolomic data were normalized by defining the median of each protein/metabolite value equal to 1.00, while missing values (if any) were filled with the observed minimum value (Chen et al., 2019). Hierarchical clustering with average linkage using Pearson correlation as a distance metric was conducted using the Mev (MultiExperiment Viewer, 4.8) software. The categories of identified proteins were determined using the online PANTHER (protein annotation through evolutionary relationship) classification system (www.pantherdb.org). A multi-omics data analysis tool, OmicsBean (http://www.omicsbean.com), was used for bioinformatics analyses including Gene Ontology (GO) analysis, KEGG pathway, and protein-protein interaction network analysis. Before correlation analysis, proteins and metabolites with the same values in more than ten samples were filtered out due to their obviously high correlations. Pearson’s product-moment correlation analysis was conducted using R statistical software. The corresponding *p*-values were calculated using the cor.test function. The calculated *p*-values were accordingly adjusted to control the false discovery rate (FDR) (Rao et al., 2014). The graphical presentations of correlations were composed with Cytoscape version 3.4.0.

## Results

### The Proteomics Characterization of Advanced Colorectal Cancer Serums

**FIGURE 1:**
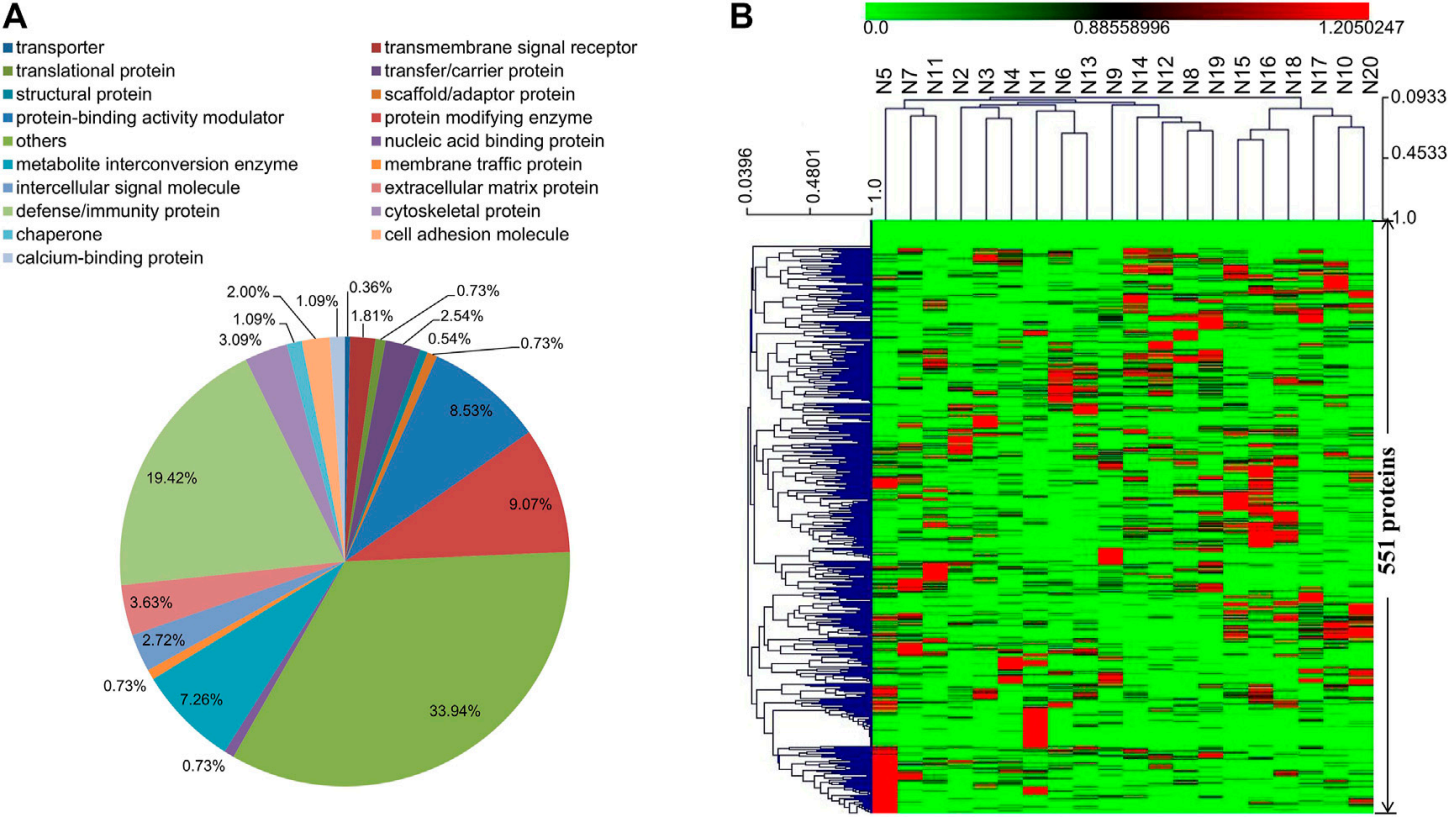
**(A)** Functional classification of the identified 551 proteins in serum samples (N1–N20) using the PANTHER classification system (www.pantherdb.org). **(B)** Hierarchical clustering analysis of the identified 551 proteins in serum samples (N1–N20).

A total of 551 proteins were identified in the DIA proteomics analysis, and the majority of them are defense/immunity proteins, protein modifying enzymes, protein-binding activity modulators, and metabolite interconversion enzymes (Supplementary Table S1 and Figure 1A). Other types of identified proteins include extracellular matrix proteins, signaling molecules, intercellular signal molecules, transmembrane signal receptors, transfer/carrier proteins, and cell adhesion molecules. Except for 25 proteins which had low abundances in all samples, the rest 526 proteins displayed remarkable changes across the 20 tested samples as illustrated in the heat-map of hierarchical clustering analysis (Figure 1B). On one hand, the enrichment of certain proteins seemed to be sample-specific. For example, 34 proteins at the bottom of the heat-map including collectin-10, lithostathine-1-alpha, and osteopontin were abundant only in sample N5. On the other hand, several proteins were specifically enriched in one or more samples. For instance, the levels of Immunoglobulin kappa variable 1–39 and C-C motif chemokine 18 were higher in samples N15 and N16 than in other samples.

The identified 551 proteins were further annotated according to GO database (Supplementary Figures S1, S2, S3). Most of the annotation terms contain cellular process, response to stimulus, and biological regulation. The main terms for cellular process include cellular anatomical entity, intracellular, and protein-containing complex. The major molecular function terms were binding, catalytic activity, and molecular function regulartor. To better understand the biological functions and/or interactions of the identified 551 proteins, we also carried out pathway annotation analysis in KEGG and mapped 251 proteins to 189 pathways. The top fifteen proteins are in complement and coagulation cascades, PI3K-Akt signaling pathway, and pathways in cancer (Supplementary Table S2).

### The Metabolomics Characterization of the Advanced Colorectal Cancer Serum Samples

**FIGURE 2:**
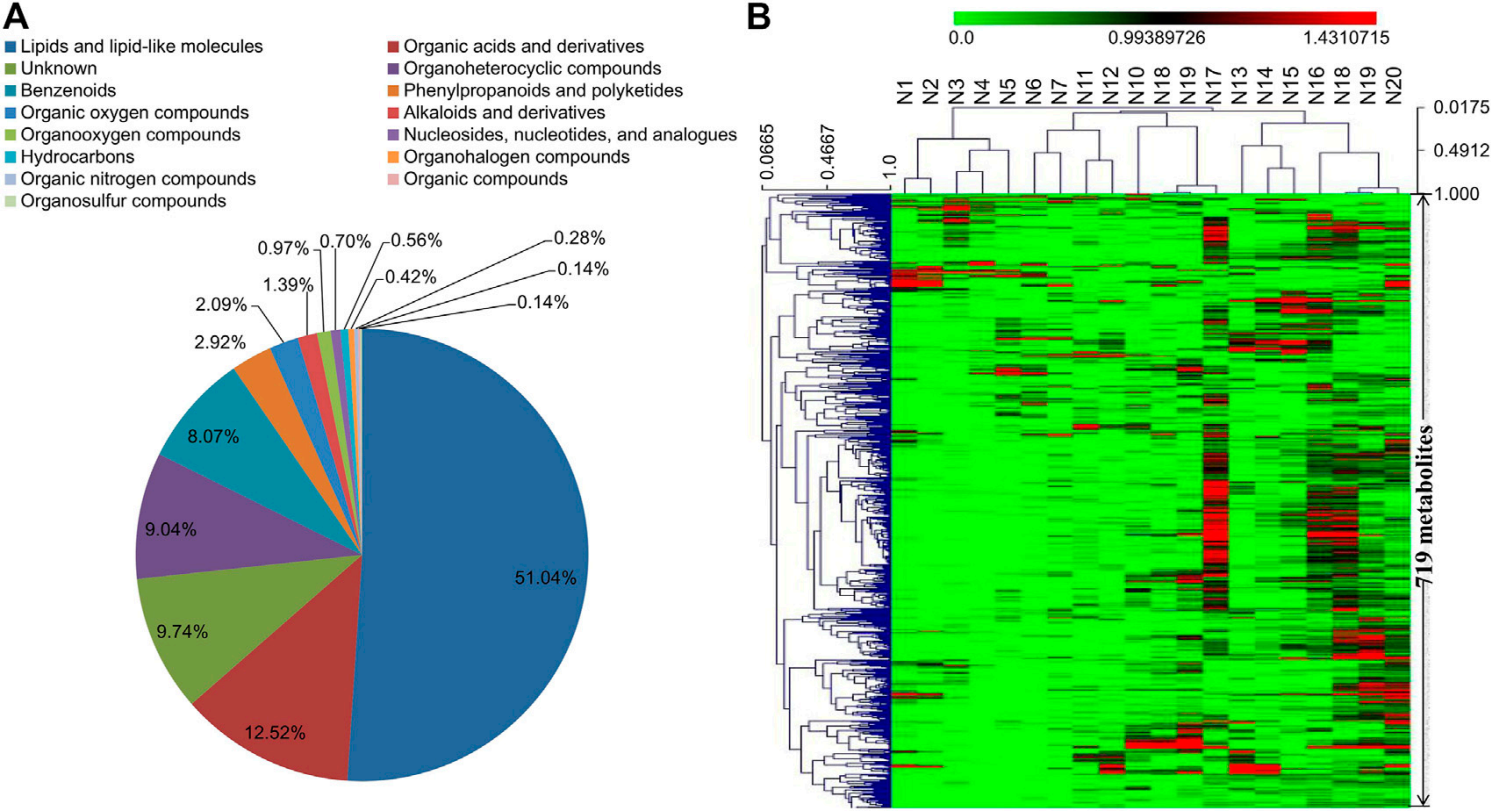
**(A)** Category information of the 719 metabolites identified in serum samples (N1–N20) according to the database from HMDB. **(B)** Hierarchical clustering analysis of the identified 719 metabolites in serum samples (N1–N20).

The same 20 serum samples analyzed by proteomics study were also subjected to non-targeted UHPLC-Q-TOF-MS/MS metabolomics analysis. A total of 9,193 positive-mode and 7,571 negative-mode ion features were detected. Based on MS/MS spectrum matching, 567 metabolites were determined in positive-mode and 431 in negative-mode data (Supplementary Tables S3, S4). Eventually, 719 non-redundant metabolites were identified in the CRC serums, and they can be classified into 15 categories according to the HMDB database (Figure 2A). Among these 719 metabolites, about 51% are lipids and lipid-like molecules. The hierarchical clustering analysis result of the metabolomics data indicated that CRC serum metabolites have less changes in their abundances than proteins in the 20 tested samples. In our previous study using six pooled samples from healthy blood donators for integrative proteomics and metabolomics analyses, similar results that serum metabolites exhibit less change than proteins were also observed (Supplementary Figure S4) (Chen et al., 2020; Deng et al., 2020). The 719 metabolites were mapped to 135 KEGG pathways, and the top three pathways with most metabolites are metabolic pathways, glycerophospholipid metabolism, and biosynthesis of amino acids (Supplementary Tables S5, S6).

### Protein-Protein Interaction Network Analysis

**FIGURE 3:**
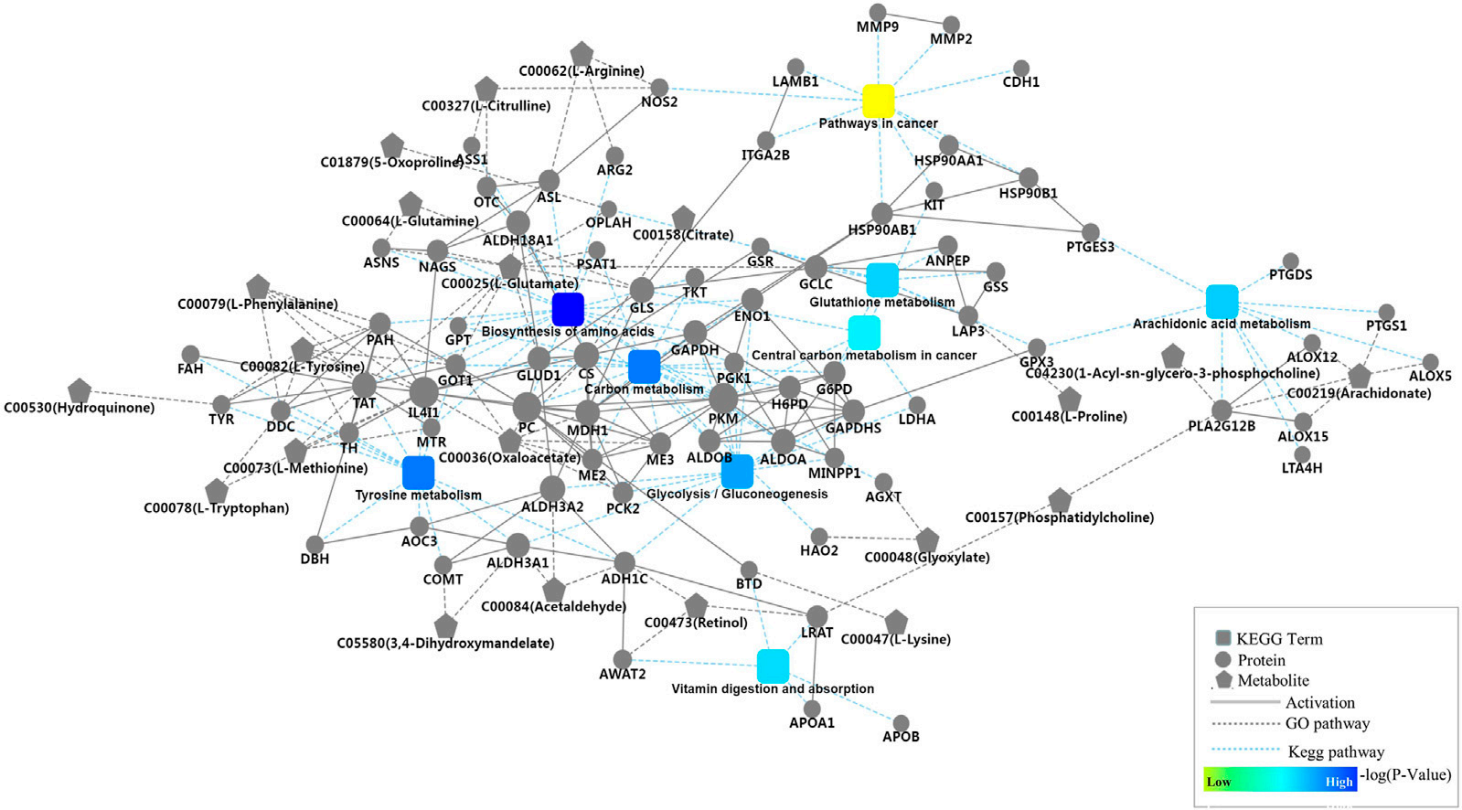
Protein-protein interaction network analysis based on nine key pathways associated with cancer cell metabolism, which involved glycolysis/gluconeogenesis, carbon metabolism, protein digestion and absorption, biosynthesis of amino acids, glutathione metabolism, vitamin digestion and absorption, central carbon metabolism in cancer, arachidonic acid metabolism, and tyrosine metabolism, as well as 80 proteins and 21 metabolites.

The 189 mapped pathways in the proteomics analysis and the 135 mapped pathways in the metabolomics analysis have 69 pathways in common, including 238 proteins and 187 metabolites in the 69 pathways (Supplementary Table S7). Ten of the 69 pathways are associated with cancer metabolisms including pathways in cancer, glycolysis/gluconeogenesis, carbon metabolism, protein digestion and absorption, biosynthesis of amino acids, glutathione metabolism, vitamin digestion and absorption, central carbon metabolism in cancer, arachidonic acid metabolism, and tyrosine metabolism. Protein-protein interaction network analysis was conducted based on the ten pathways to provide further insight into the developmental and physiological processes underlying advanced CRC. All of the ten pathways except protein digestion and absorption were covered in the network with 80 proteins and 21 metabolites (Figure 3). The 80 proteins were mapped to corresponding pathways via KEGG analysis, and the metabolites were connected to proteins *via* GO database annotations. Two pathways, biosynthesis of amino acids and carbon metabolism, dominate the network and include a large number of proteins such as PKM, GAPDH, ALDOA, and ALDOB that plays important roles in cellular proliferation. Other pathways such as glutathione metabolism, tyrosine metabolism, and glycolysis/gluconeogenesis in the network generate key products that promote cell survival and growth. Arachidonic acid metabolism pathway in the network consists of nine detected proteins, and this pathway play important roles in the development of various cancers (Borin et al., 2017).

### Correlation Analysis Between the Detected Proteins and Metabolites

**FIGURE 4:**
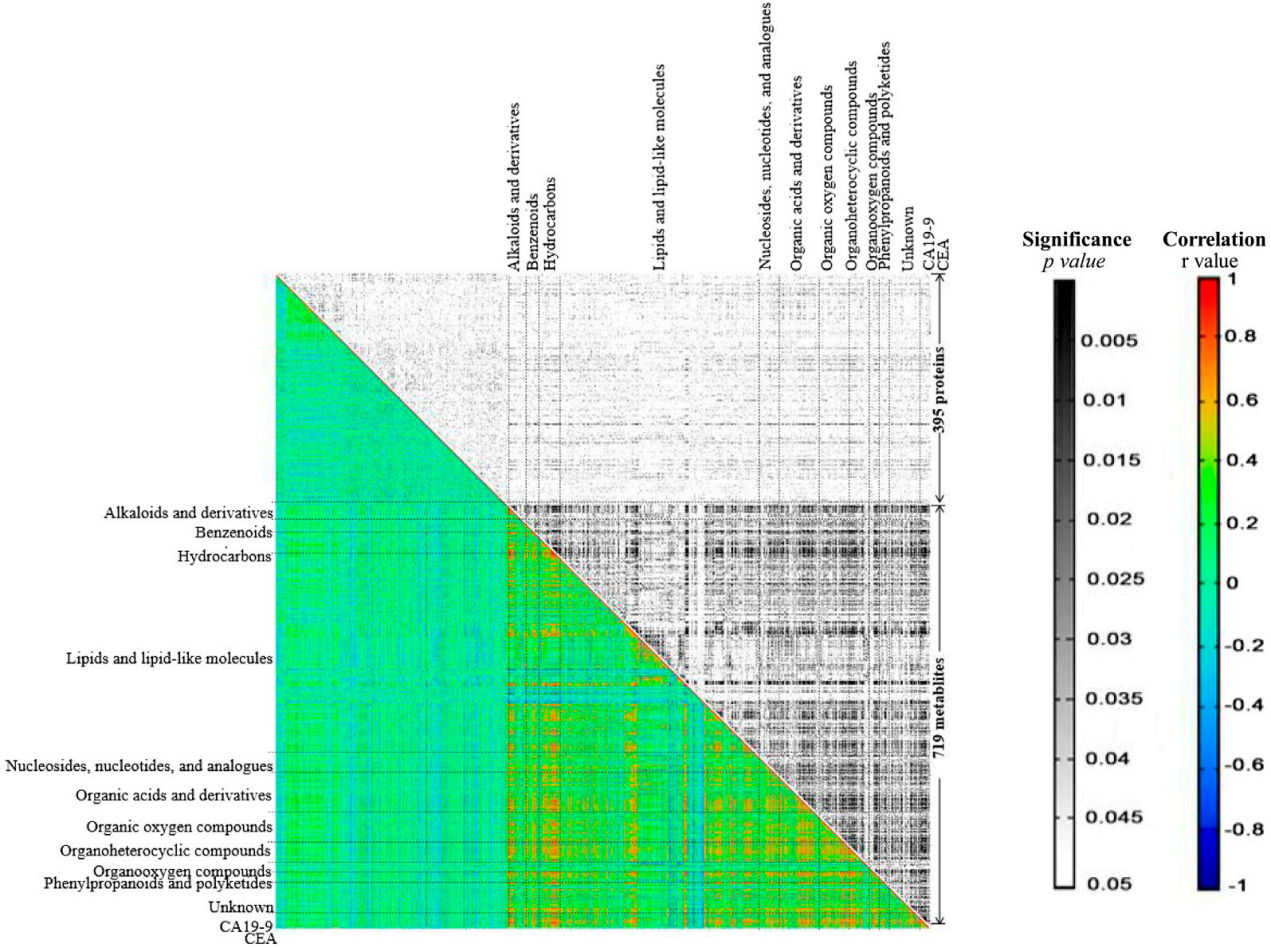
The heat-map generated from correlation analysis. *X* and *Y*-axes were categorized into proteins/metabolites/CA19-9/CEA. In the black and white area rectangles represent *p*-values resulting from Pearson correlation coefficient, while in the colored area rectangles represent *r* values respective to Pearson correlation coefficient computation.

**FIGURE 5:**
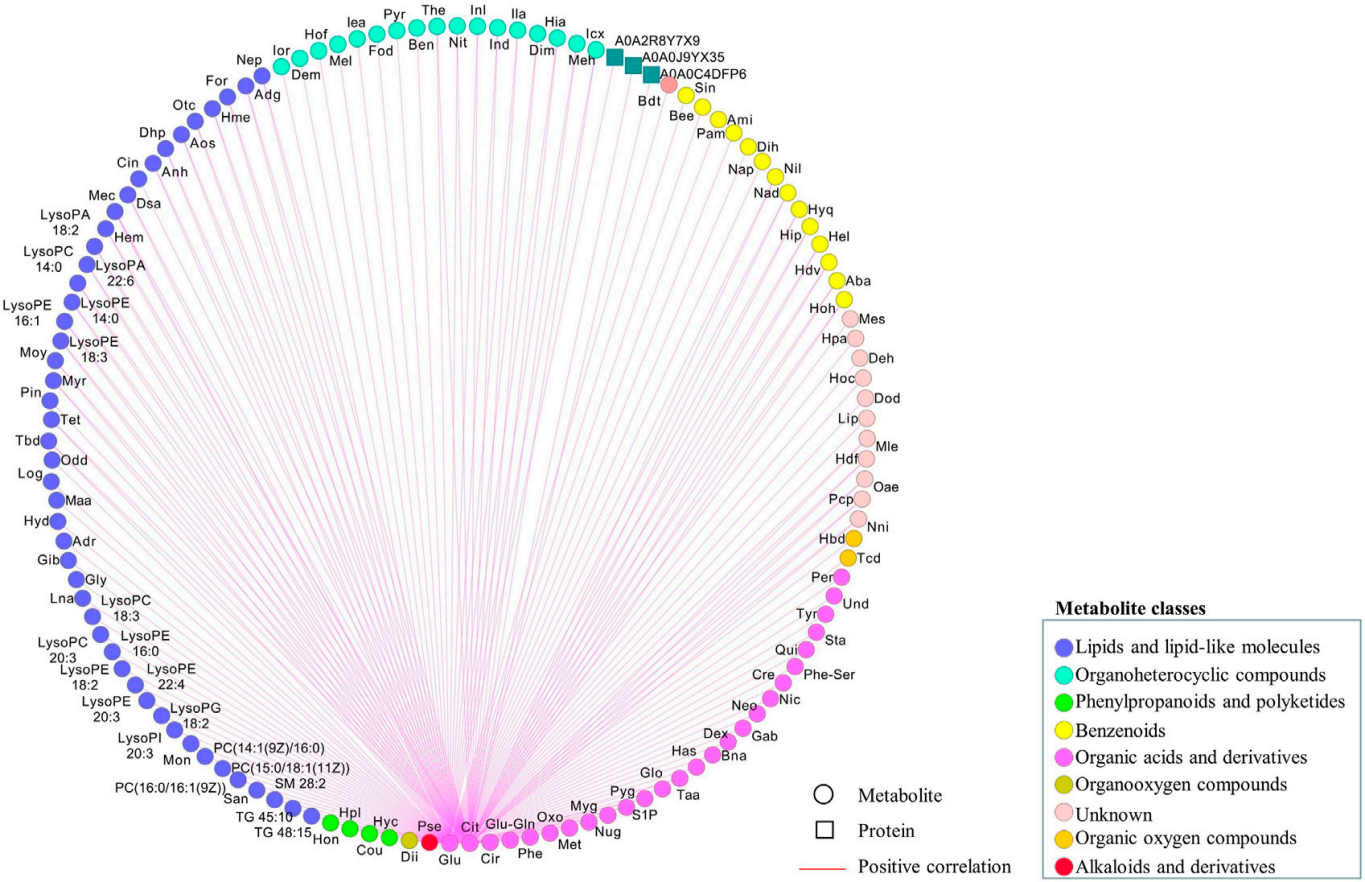
Regulatory network associated with citric acid or glutamine based on significant correlations (*r* ^2^ ≥ 0.49 and FDR ≤0.05). Metabolites and proteins were respectively represented as circular and rectangle, and their relations as edges. Metabolites categorized into different pathways were displayed in different node colors. The positive correlations were shown in red. Computations of the correlations were performed under the R environment. Cytoscapewas employed to generate the graphical output of the networks.

To further explore the regulatory network in advanced CRC, network-based analysis (Rao et al., 2014) was conducted to analyze the correlations among the identified proteins and metabolites (Supplementary Table S8), as well as their correlations with two tumor markers, CEA and CA 19–9. Pearson pair-wise correlation of 1116 elements including 395 proteins, 719 metabolites and the two tumor markers were calculated, and the results were presented in a heat-map displaying a total of 622,170 correlations with scores ranging from −0.8978 to 0.9991 (Figure 4). There were 23,201 significant correlations with *r* ^2^ ≥ 0.49 and FDR ≤0.05. Among them, 22,891 were positive correlations and the rest 310 were negative ones. There were much more significant correlations between metabolites than those between proteins or with tumor markers. Lipids and lipid-like metabolites dominated the significant correlations, accounting for nearly 63% of all significant correlations. These significant correlations were not observed in a previous study on the healthy control serums (Supplementary Figure S5) (Chen et al., 2020; Deng et al., 2020). Two important metabolites, citric acid and glutamine, are positively and significantly correlated (Supplementary Table S9), and other correlations with citric acid and glutamine were shown in Figure 5. There were 120 positive and significant correlations with glutamine and 63 positive and significant correlations with citric acid. In total, there were 182 positive and significant correlations between citric acid/glutamine and 126 molecules, and most of the 126 molecules were lipids and lipid-like metabolites. Other molecules which had positive and significant correlations with citric acid or glutamine include 24 organic acids and their derivatives, 14 benzenoids, 17 organoheterocyclic compounds, and three proteins. There were 18 positive and significant correlations between ten elements (five metabolites and five proteins, Table 2) and the two cancer marks (CEA and CA 19–9). The correlation between CEA and CA 19–9 was also very strong. In the previous study using six pooled healthy blood samples, there was no significant correlation between CEA and CA 19–9, and there was no significant correlation between the above mentioned ten elements and the two cancer markers (Supplementary Figure S5) (Chen et al., 2020; Deng et al., 2020).

**TABLE 2:** The list of significant correlations associated with CEA or CA 19–9.

Protein/metabolite 1	Protein/metabolite 2	Correlation coefficient	*p* value
CEA	Hydroquinone	0.74	1.99E-04
CEA	CA19-9	0.96	1.46E-11
CEA	Immunoglobulin heavy variable 1–69D	0.74	2.18E-04
CA19-9	Immunoglobulin lambda variable 4–60	0.78	4.22E-05
CEA	Immunoglobulin lambda variable 4–60	0.82	1.00E-05
CEA	Immunoglobulin kappa variable 2–40	0.71	4.84E-04
CA19-9	16beta-16-Hydroxy-3-oxo-1,12-oleanadien-28-oic acid	0.71	4.82E-04
CEA	16beta-16-Hydroxy-3-oxo-1,12-oleanadien-28-oic acid	0.73	2.50E-04
CA19-9	SM 28:3; SM(d14:2/14:1)	0.76	9.02E-05
CEA	SM 28:3; SM(d14:2/14:1)	0.79	3.42E-05
CA19-9	Coagulation factor XIII A chain	0.75	1.37E-04
CEA	Coagulation factor XIII A chain	0.82	1.16E-05
CA19-9	Leucenol	0.72	3.40E-04
CEA	Leucenol	0.76	9.88E-05
CEA	Plasma kallikrein (fragment)	0.70	5.39E-04
CA19-9	Plasma kallikrein (fragment)	0.74	1.73E-04
CA19-9	1-(1′,3′-Benzodioxol-5′-yl)-2-butanamine	0.81	1.34E-05
CEA	1-(1′,3′-Benzodioxol-5′-yl)-2-butanamine	0.87	8.56E-07

## Discussion

CRC is one of the most common and lethal cancers worldwide, but has poor diagnosis and few effective treatment options, especially for patients with advanced CRC. These limitations highlight the importance of gaining new understanding of the pathogenesis of advanced CRC. We employed an integrated proteomics and metabolomics strategy to investigate the serum samples from 20 CRC patients, including 17 diagnosed with stage IV CRC and three with stage III advanced CRC. The ages of the 20 patients ranged from 29 to 76. The 20 patients had various types of advanced CRC including sigmoid colon cancer, right colon cancer, rectal cancer, ascending colon cancer, and adenocarcinoma of the junction of rectum and sigmoid. The study was a multi-omics one consisting of both proteomics and metabolomics investigations on each serum sample, and this enabled us to explore both proteome and metabolome changes in the serums of advanced CRC patients and the interactions between the proteome and metabolome.

Experimental human body fluid samples include blood, breast milk, tears, urine and malignant pleural efusions, etc., among which blood is the most commonly adopted one for discovering new biomarker to predict treatment effects and prognosis of diseases including cancers (Deng et al., 2020). Blood can be noninvasively collected in large quantity through a simple procedure, and changes in blood proteome and metabolome can reflect physical or pathological disturbances to an otherwise balanced and homeostatic system (Zhou et al., 2019). In the present study, DIA-MS and UPLC/Q-TOF-MS/MS technologies were used to investigate proteomes and metabolomes of advanced CRC serum samples. The proteomics study detected a total of 551 proteins, most of which are defense/immunity proteins, protein modifying enzymes, and protein-binding activity modulators. Other types of detected proteins include metabolite interconversion enzymes, extracellular matrix proteins, and signaling molecules. A total of 719 named metabolites were determined in the metabolomics study, and 649 of them can be classified into 14 categories such as lipids and lipid-like molecules, organic acids and derivatives, and organoheterocyclic compounds, etc. These 649 metabolites cover most of the central metabolism pathways such as carbohydrate super pathway, amino acid super pathway, lipid super pathway, and nucleotide super pathway. Compared to previous reports, the current study revealed changes of more serum proteins and metabolites related to advanced CRC, which is helpful in uncovering more molecular changes and selecting novel biomarkers.

The KEGG pathway analysis revealed that the detected proteins and metabolites share 69 common pathways, and ten of them were associated with cancer cell metabolisms including glycolysis/gluconeogenesis, biosynthesis of amino acids, glutathione metabolism, and arachidonic acid metabolism. We used OmicsBean online software to construct a protein-protein interaction network in advanced CRC serum. This network covers nine cancer-associated pathways, 80 proteins and 21 metabolites. Metabolic reprogramming, as a hallmark of cancer, has become a hot topic in cancer research over the past decade (Koppenol et al., 2011). The well documented Warburg effect is characterized by an increase in glucose uptake and lactate production. There are 13 detected proteins in glycolysis/gluconeogenesis pathway (Figure 3), and most play important roles in the development of cancers including CRC (Gimm et al., 2001; Patel et al., 2008; Tsai et al., 2010; Duell et al., 2012; Ahmad et al., 2013; Cui et al., 2014; Leithner et al., 2015; Yun et al., 2015; Dayton et al., 2016; He et al., 2016; Dai et al., 2018). For example, phosphoglycerate kinase 1 (PGK1) is reported to be a promoter of metastasis in CRC, and high expression of ALDOA is associated with poor CRC prognosis.

Glutathione metabolism plays both beneficial and pathogenic roles in a series of malignancies (Bansal and Simon, 2018). In our study, eight proteins including G6PD, GPX3, and LAP3 which are reported to be involved in colon cancer cell growth were connected to glutathione metabolism (Pelosof et al., 2017; Zhang et al., 2017; Yang et al., 2018). Nine proteins in the constructed network are associated with arachidonic acid metabolism, suggesting that arachidonic acid pathway may play important roles in advanced CRC. Many studies have demonstrated the connection between arachidonic acid metabolism and carcinogenesis (Hong et al., 2004). Habermann et al. reported that SNPs inside PTGS1, ALOX5, ALOX12, and ALOX1 affect fatty acid metabolisms in CRC (Habermann et al., 2013). In addition, 21 metabolites including citrate, oxaloacetate, arachidonate and nine standard amino acids were connected to the nine cancer-associated pathways via related proteins.

A regulatory network to reveal key regulatory elements in advanced CRC was also constructed by correlation analysis (Rao et al., 2014). A large number of significant correlations were discovered, most of which were positive correlations. The highly positive associations between every two metabolites suggested the conserved roles of metabolome in the human serum, which was in line with the observation in hierarchical clustering analysis. Lipids and lipid-like molecules dominate the significant correlations, suggesting their essential roles in advanced CRC (Beloribi-Djefaflia et al., 2016). In the metabolomics studies of aqueous humor samples from patients with high myopia and various mature seeds including maize kernels, the identified amino acids have conserved roles, which are totally different from the findings in the current study (Toubiana et al., 2012; Rao et al., 2014; Ji et al., 2017). Most of the significant correlations with citric acid and glutamine are also correlated to lipids and lipid-like molecules. There were 18 positive and significant correlations to CEA or CA 19–9. CEA and CA19-9 are acknowledged markers for diagnosing early stages of CRC and predicting treatment effect, but with limitations (Hissong and Pittman, 2020). In the present study, CEA and CA 19–9 are strongly correlated, but the levels of CEA and CA 19–9 exceeded the standard reference values in only 55% of the patients. Five metabolites and five proteins with strong and positive correlations with CEA or CA 19–9 have been demonstrated to be potential biomarkers involved in modulating cancer cell growth (Byeon et al., 2018). For example, hydroquinone was determined to be able to increase skin cancer risk, while the biosynthesis of sphingomyelin was reported to modulate cancer cell death and growth (Lewis et al., 2018). Kulp et al. found that mimosine can block cell cycle progression in asynchronous human breast cancer cells by chelating irons (Kulp and Vulliet, 1996). Activation peptide of the coagulation factor XIII (AP-F13A1) and plasma kallikrein (fragment) were identified to be novel biomarkers for the screening of CRC and lung cancer, respectively (Chee et al., 2008; Peltier et al., 2018). Together with CEA and CA 19–9, these three metabolites (hydroquinone, sphingomyelin and mimosine and) and two proteins (coagulation factor XIII A chain and plasma kallikrein) are potential biomarkers, to improve the accuracy of diagnosis and monitoring of CRC. These potential new biomarkers need to be validated in further studies with more patients and controls.

## Conclusion

In summary, the present study reported an integrative proteomics and metabolomics investigation of advanced CRC serums. The constructed protein-protein interaction network and correlation analysis revealed key regulatory elements and pathways in advanced CRC, and new potential biomarkers for diagnosis and monitoring of CRC were selected. Since the number of patients in the present study was limited, future validation studies need to be conducted to validate the discoveries reported in this study.

## Data Availability

The datasets presented in this study can be found in online repositories. The names of the repository/repositories and accession number(s) can be found in the article/Supplementary Material.
