# Supplementary material for: Molecular Characterization of Advanced Colorectal Cancer Using Serum Proteomics and Metabolomics
Source: Front Mol Biosci. 2021 Jul 27;8:687229. doi: 10.3389/fmolb.2021.687229 (PMC8353147; doi:10.3389/fmolb.2021.687229)
Supplement: Supplementary file 1 [file Presentation1.PPTX]

## Slide 1
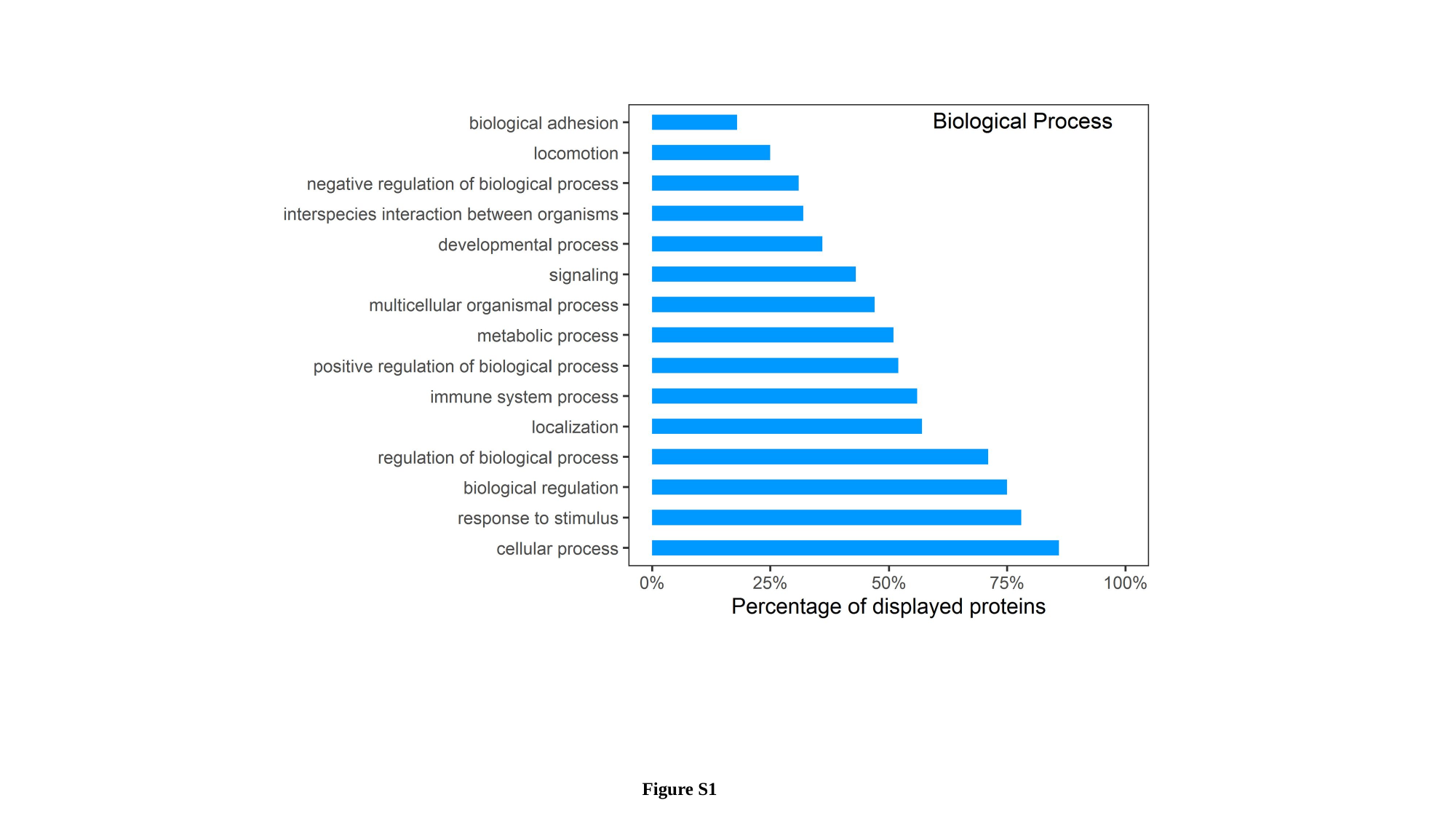

Figure S1

## Slide 2
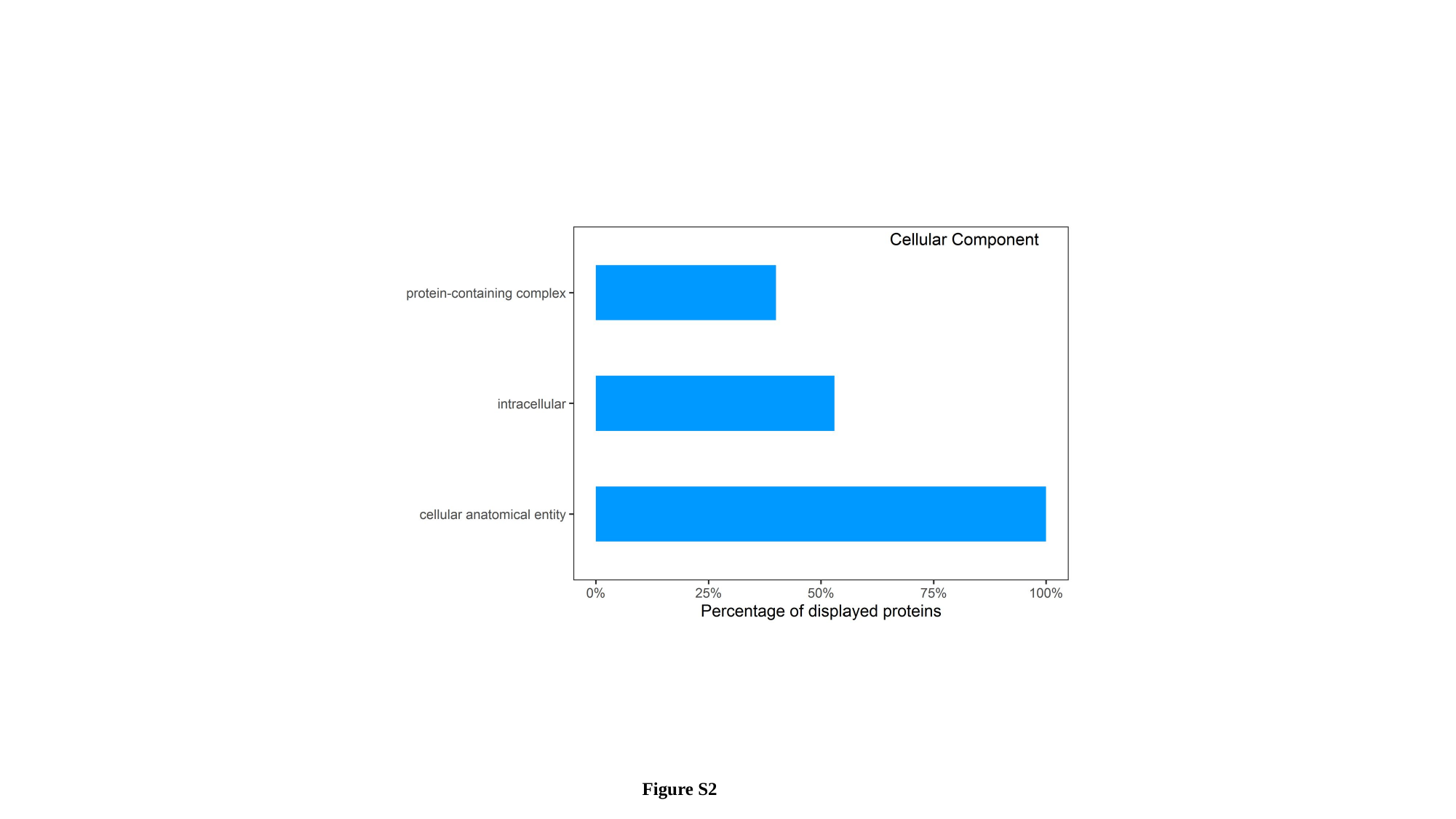

Figure S2

## Slide 3
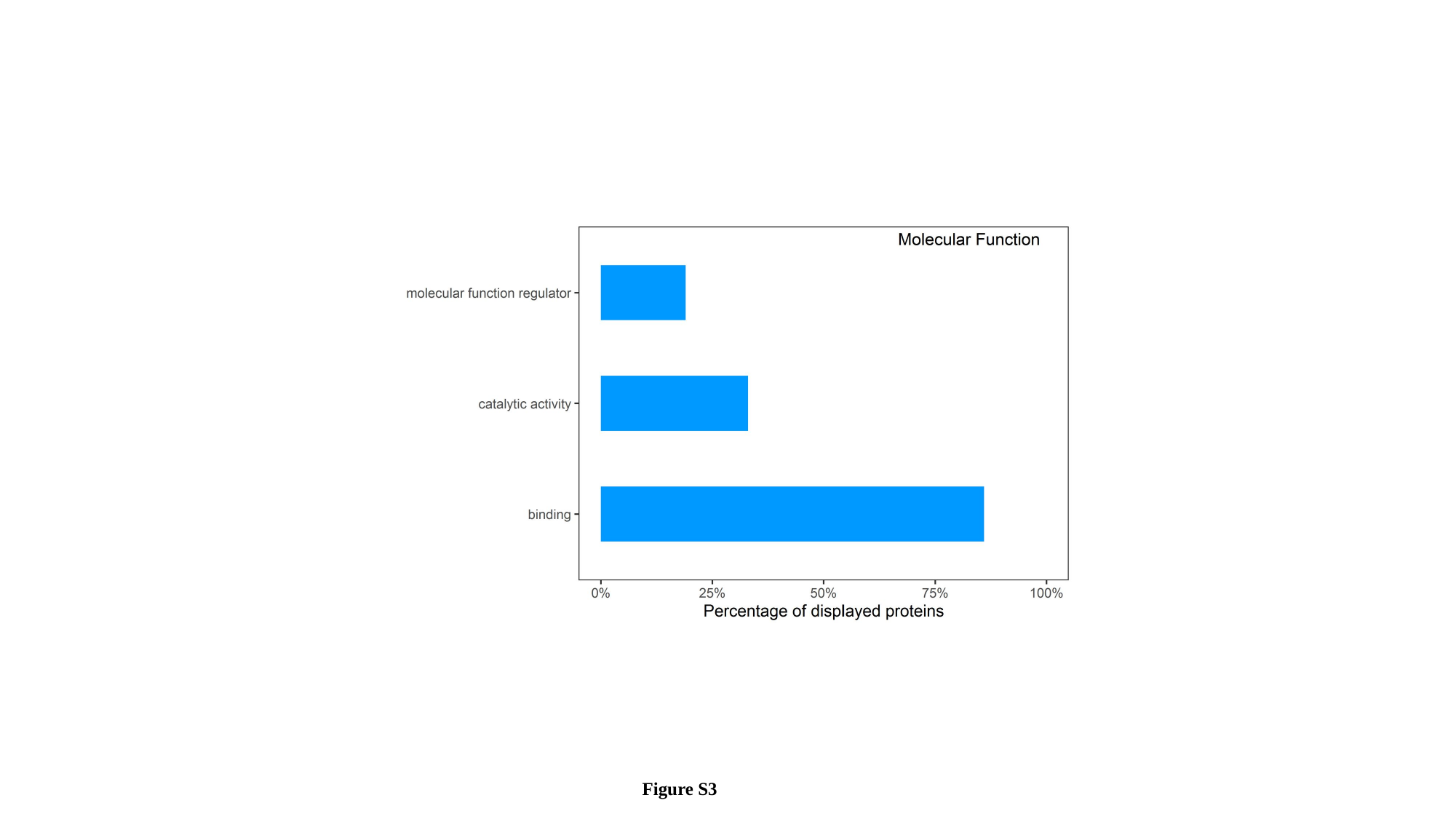

Figure S3

## Slide 4
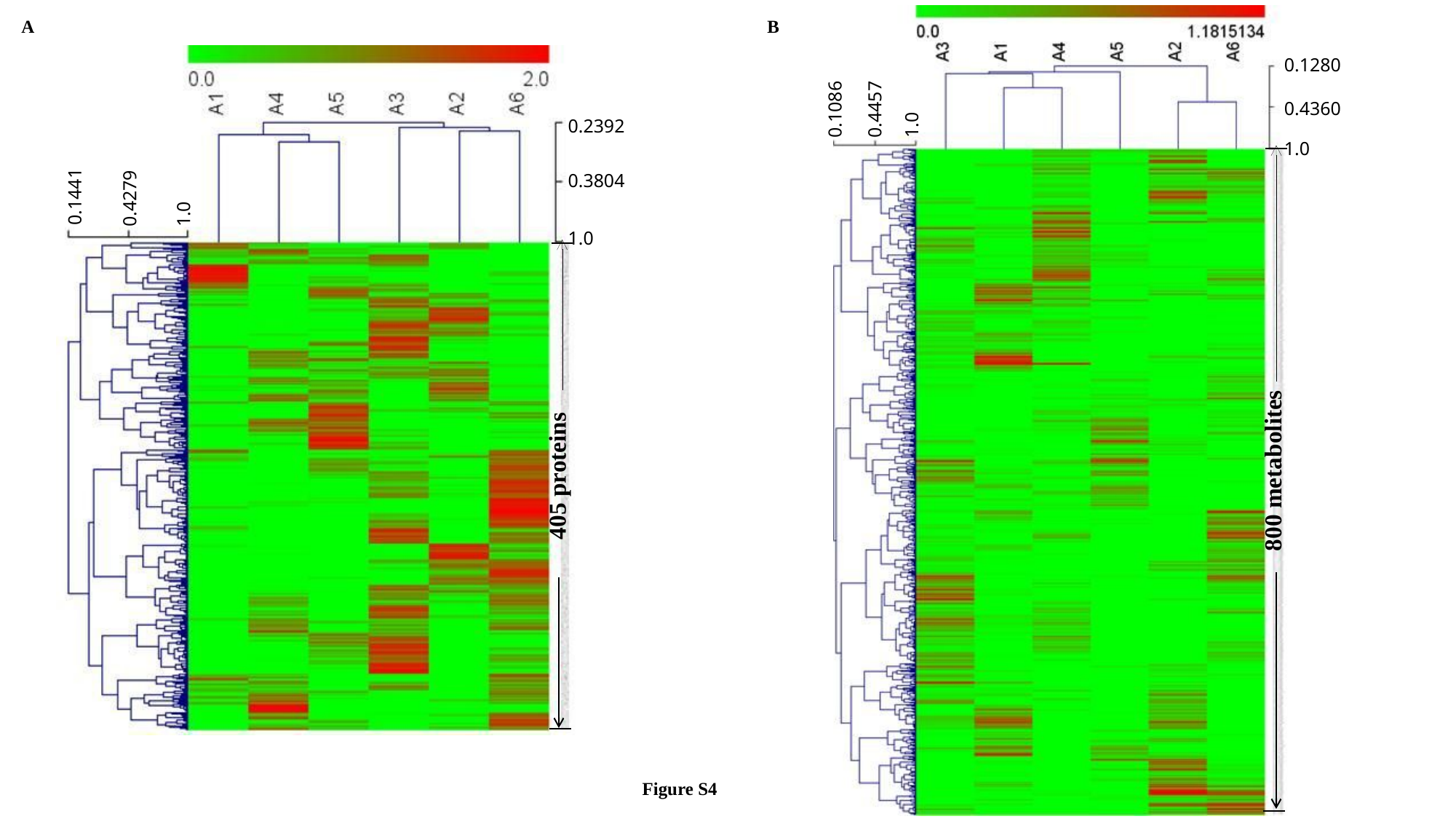

A
B
0.1086
0.4457
1.0
0.1280
0.4360
1.0
0.2392
0.3804
1.0
0.1441
0.4279
1.0
800 metabolites
405 proteins
Figure S4

## Slide 5
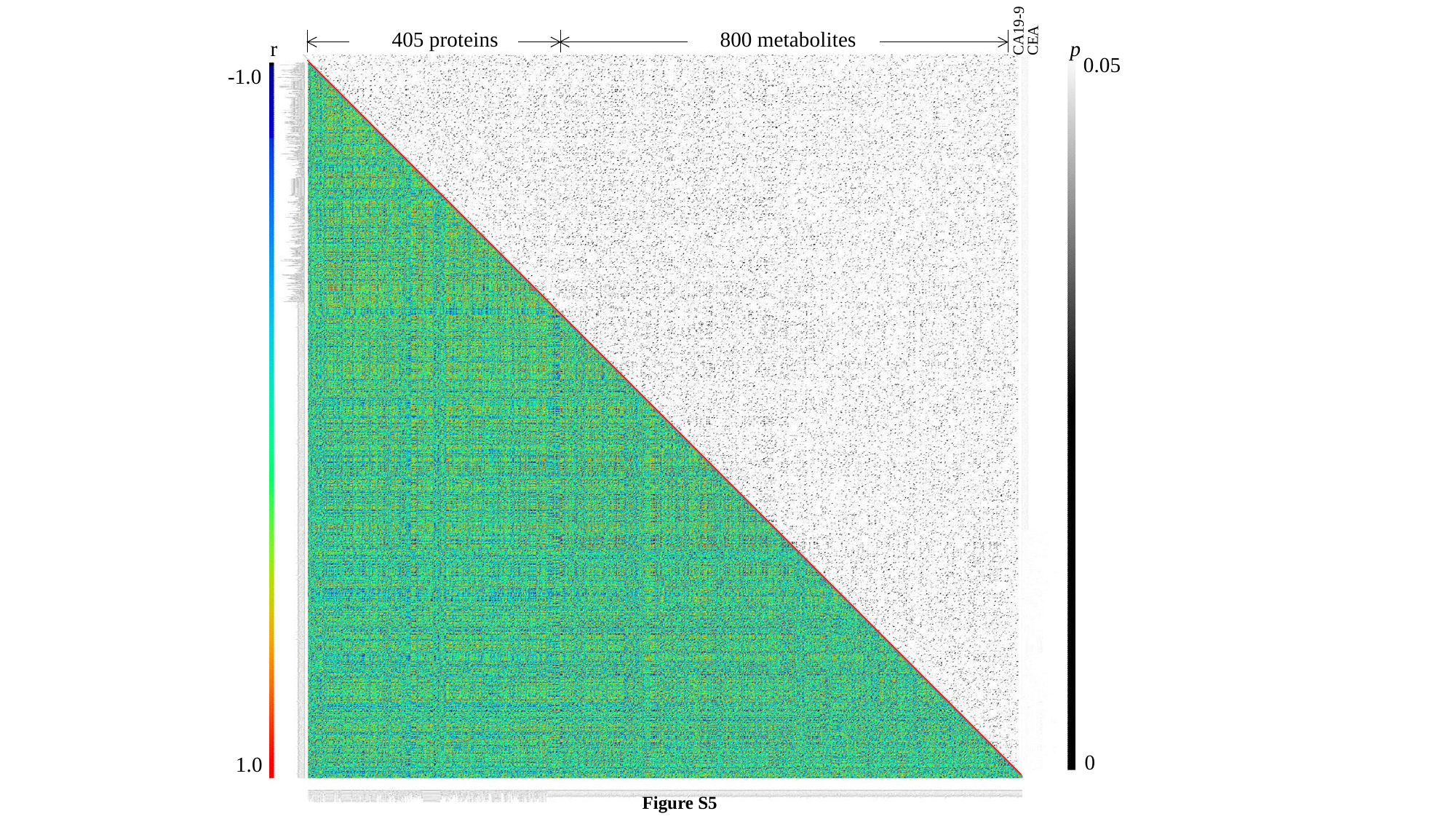

r
p
800 metabolites
405 proteins
0.05
-1.0
1.0
0
CA19-9
CEA
Figure S5
